# Supplementary material for: Carbon monoxide metabolism in freshwater anaerobic methanotrophic archaea
Source: Nat Commun. 2026 Apr 14;17:3460. doi: 10.1038/s41467-026-70080-4 (PMC13079737; doi:10.1038/s41467-026-70080-4)
Supplement: Supplementary file 1 — Supplementary Information [file 41467_2026_70080_MOESM1_ESM.pdf]

Supplementary Information: Carbon monoxide metabolism in freshwater anaerobic methanotrophic archaea

Reinier A. Egas[1], Heyu Lin[2], Andy O. Leu[2], Gene W. Tyson[2], Simon J. McIlroy[2] and Cornelia U. Welte[1]

[1] Department of Microbiology, Radboud Institute for Biological and Environmental Sciences, Radboud University, Heyendaalseweg 135, 6525AJ Nijmegen, The Netherlands

[2] Centre for Microbiome Research, School of Biomedical Sciences, Queensland University of Technology (QUT), Translational Research Institute, Woolloongabba, QLD, Australia

Supplementary Table 1. Designed primers to confirm circularity and validate the transitions between unique and shared regions of identified mobile genetic element in this study. Primers were manually designed to amplify the two junctions. Primer pairs targeted the left junction (LJ-F/R, positions 64149 - 64843) and right junction (RJ-F/R positions 91243 - 91892).

**LJ-MGE_F**	
**Sequence (5'→3'):**	**TTT CTT CGC ACA CTT GCT TC**
Reverse Complement (5'→3'):	GAA GCA AGT GTG CGA AGA AA
Reverse Sequence (5'→3')	CTT CGT TCA CAC GCT TCT TT
Complement Sequence (5'→3'):	AAA GAA GCG TGT GAA CGA AG
Base Composition:	A x 2, C x 7, G x 2, T x 9, U x 0, Wobbles x 0
GC Content:	45 %
Melting Temperature (TM):	55.3 °C
**LJ-MGE_R**	
**Sequence (5'→3'):**	**TGC CGC TTT TTT TAC TGT TG**
Reverse Complement (5'→3'):	CAA CAG TAA AAA AAG CGG CA
Reverse Sequence (5'→3')	GTT GTC ATT TTT TTC GCC GT
Complement Sequence (5'→3'):	ACG GCG AAA AAA ATG ACA AC
Base Composition:	A x 1, C x 4, G x 4, T x 11, U x 0, Wobbles x 0
GC Content:	40 %
Melting Temperature (TM):	53.2 °C
**RJ-MGE_F**	
**Sequence (5'→3'):**	**ACA ATC CGC CCC CAC TAA TC**
Reverse Complement (5'→3'):	GAT TAG TGG GGG CGG ATT GT
Reverse Sequence (5'→3')	CTA ATC ACC CCC GCC TAA CA
Complement Sequence (5'→3'):	TGT TAG GCG GGG GTG ATT AG
Base Composition:	A x 6, C x 10, G x 1, T x 3, U x 0, Wobbles x 0
GC Content:	55 %
Melting Temperature (TM):	59.3 °C
**RJ-MGE_R**	
**Sequence (5'→3'):**	**TTT GCC AGT TCA TCT CGC C**
Reverse Complement (5'→3'):	GGC GAG ATG AAC TGG CAA A
Reverse Sequence (5'→3')	CCG CTC TAC TTG ACC GTT T
Complement Sequence (5'→3'):	AAA CGG TCA AGT AGA GCG G
Base Composition:	A x 2, C x 7, G x 3, T x 7, U x 0, Wobbles x 0
GC Content:	52.6 %
Melting Temperature (TM):	56.7 °C

Supplementary Figure 1. Multiple sequence alignment of Ni-CODH proteins identified in this study. Twelve high-confidence Ni-CODHs were extracted from metagenome-assembled genomes (MAGs) in which all key residues of the D-cluster, B-cluster, acid-base catalytic site, and C-cluster were preserved. Residues involved in metal cluster coordination and catalysis are highlighted: D-cluster (green), B-cluster (orange), acid-base catalytic residues (blue), and C-cluster (pink). Two MAG-derived Ni-CODHs with incomplete complete cluster motifs are displayed but excluded from further analysis. PADDDONK_02117 encodes CdhA, which lacks a conserved D-cluster. Identifiers: GIHMANB, Mobile genetic element; PADDDONK, '*Ca.* Methanoperedens BLZ2'; AKAIOEBN, o_Aggregatilineales s_SB15 sp008933735; EPCNEHLO, o_Aggregatilineales s_CFX10 sp019913005; GLJNLELP, o_Acetivibrionales g_MnB-11; HFBOOHMF: o_Brocadiales g_Brocadia.

D-cluster; **B-cluster**; **acid-base catalysis**; **C-cluster**

```
                ....|....| ....|....| ....|....| ....|....| ....|....| ....|....| ....|....| ....|....|
                     5         15         25         35         45         55         65         75
GIHMDANB_00018  MKENASVHKS TLEMYEFDRY NAQQPCTYGA QGICCQLCSH GPCRITKATV GICGATADVI AARNFLRLTA GGSAAYTHHL
GIHMDANB_00023  MPEDVSAHLS VIQMHKFDRA LAQGKCSFCE QGISCQLCSN GPCRIKGVDR GVCGIDADAM AMRNMMFLNT MGIAAYTYHA
PADDDONK_01837  MKENASVHKS TLEMYEFDRY NAQQPCTYGA QGICCQLCSH GPCRITKATV GICGATADVI AARNFLRLTA GGSAAYTHHL
PADDDONK_01842  MPEDVSAHLS VIQMHKFDRA LAQGKCSFCE QGISCQLCSN GPCRIKGVDR GVCGIDADAM AMRNMMFLNT MGIAAYTYHA
PADDDONK_02117  M-EIQTPRPE IIGLRDWDYR IIDRYPVYTP TGDTCDFCTY GKCDLTGNKE GACGIDLEGQ SARQALMVSI MGAACHSAHG
PADDDONK_03833  MGEIKNPDNA TDEMIRFHRN GNKKACNFGS RGICCKQCML GPCRISRSLK GTCGASADTI VARNLLMMIG RGTAAHSSHA
PADDDONK_03053  MNEIKNPDNA TDEMIRFHRN GNKKACNFGS RGICCKQCML GPCRISRSLK GTCGASADTI VARNLLMMIG RGTAAHSSHA
PADDDONK_03117  M-ESVSAHES VIKMHNFDRA QAQGKCSFCE QGISCQLCSN GPCRIKGAER GVCGIDADAM AMRNLMFLNT MGTAIYTYHA
AKAIOEBN_03183  MPEEYSIDPA AQQMLIFTRA DDMVPCNIGG AGMCCKLCGM GPCRLTEGQT GVCGATIDTI QARNFIRAIA AGSAAHSDHG
ECJEKJPK_00295  ---------- ---------- ---------- ---------- ---------- ---------- ---------- ----------
ECJEKJPK_02254  ---------- ---------- ---------- ---------- ---------- ---------- ---------- ----------
EPCNEHLO_04340  MPLEQSIDPA AQEMLIFSRA DAMAPCNIGA AGMCCKMCGM GPCRLTEGQT GVCGATIDTI QARNFVRAVA AGSAAHSDHG
GLJNLELP_01063  MLNRRTVDPA ANEMLGWDRY KAQQPCKFGQ TGICCRICIQ GPCRIIGQDK GICGADVFTI VARNLVRYIA GGASAHSDHG
HFBOOHMF_00942  MEKKKSADE- --IMLDYDRY QAQLPCGYGS LALCCRHCNY GPCNIDGPKK GVCGADANTF AARHFLRMSG AGTACHSDHA

                ....|....| ....|....| ....|....| ....|....| ....|....| ....|....| ....|....| ....|....|
                     85         95        105        115        125        135        145        155
GIHMDANB_00018  EMIAKTLKAT AQGKTKIQDP ALGLDELLLE KFAPKTRLEV WKKLGGTYAE VGDAMTKTLT SIDTDPVDLL LTTLKVGIAT
GIHMDANB_00023  KEVAKTLRAT ALGKTKITDE KLGLKILSNL RFAPKSRIDL WRKLGGPLNE TTDCVTSSMT NIDGDYVSLA KKALRLGISC
PADDDONK_01837  EMIAKTLKAT AQGKTKIQDP ALGLDELLLE KFAPKTRLEV WKKLGGTYAE VGDAMTKTLT SIDTDPVDLL LTTLKVGIAT
PADDDONK_01842  KEVAKTLRAT ALGKTKITDE KLGLKILSNL RFAPKSRIDL WRKLGGPLNE TTDCVTSSMT NIDGDYVSLA KKALRLGISC
PADDDONK_02117  RHLLNYLIKK YGEDFDV-GP S-NLQPTETM GIQPKTIGDF RPVLDYVEEQ LTQLIATTNT GQEGAARDFE SKALHAGMLD
PADDDONK_03833  LHVASTLLKT VRNNTTIKEP KINCTYMRFR SYYPPDIFEN LSKLGSAGRE LLDSGHETSM GTMADPAGFV LHAARLGVAD
PADDDONK_03053  LHVASTLLKT VRNNTTIKEP KINCTYMRFR SYYPPDIFEN LSKLGSVGRE LLDSGHETSM GTMADPAEFV LHAARLGVAD
PADDDONK_03117  KSVAKTLEAT ALGKTQIKDE KMGVKVLSNL RFAPKSRIEL WRKLGGPLHE VMDSVSSTMT NVDGDYVSLA KKALRLGISC
AKAIOEBN_03183  RDMAFTLKAV AKGEAMIRDV HYNIPELAPV IRAPKKRQQL WRERRGIDRE VVEALHRTHI GDDQDPEHIL QHAIRTALAD
ECJEKJPK_00295  ---------- ---------- ---------- ---------- ---------- ---------- ---------- ----------
ECJEKJPK_02254  ---------- ---------- ---------- ---------- ---------- ---------- ---------- ----------
EPCNEHLO_04340  RDMAFTLKAV AEGKTMIRDV YYDIPHIAPI IRAPEKRQKI WQEQRGIDRE VVESLHRTHI GDDQDPEHLL QHTIRTALAD
GLJNLELP_01063  REIANTLLHA AEGHAKVTDK RIGIEECTWE TTIDEERKQK FKHTTAIDRA VVQLLHQTHM GTDADPVNII FGGLRAALAD
HFBOOHMF_00942  RAAAHLLVAT ARGEARIKDV CFGVKTLLFL KRAPESRQKI WEKLRAIDRE VTESLHRTGM GGDQDYRNLT LQAMRVALAD

                ....|....| ....|....| ....|....| ....|....| ....|....| ....|....| ....|....| ....|....|
                    165        175        185        195        205        215        225        235
GIHMDANB_00018  YMGLVATITL QDILLGTPTP TTSSADLGII DPKAVNIVAH GHVPLMATAV LRATQEMQKD IKLLGSMCTG QELMQRSATG
GIHMDANB_00023  YGSQIPLEMG QDILFGTPKP HAVNVDLGII DPAYVNIAVN GHEPFIGVAL IKAAHKNQEG LHIIGSIETG QELVQRYNIG
PADDDONK_01837  YMGLVATITL QDILLGTPTP TTSSADLGII DPKAVNIVAH GHVPLMATAV LRATQEMQKD IKLLGSMCTG QELMQRSATG
PADDDONK_01842  YGSQIPLEMG QDILFGTPKP HAVNVDLGII DPAYVNIAVN GHEPFIGVAL IKAAHKNQEG LHIIGSIETG QELVQRYNIG
PADDDONK_02117  LLGMEVADII QISCLGFPKA PMAEIGMGIL DPKKPVVICV GHNIAAPAYI LDYMDGQFDK IEIAGLCCTA HDMTRYNKAK
PADDDONK_03833  ISSLIISAEL QDVLFGTPKI FSSKIGFNVL EKDKVNVVIQ GHVPLLSEKI IEFSDLLLEG INIVGCCCTG NDALMRHGIP
PADDDONK_03053  ISSLIISAEL QDVLFGTPKI FSSKIGFNVL EKDKVNVVIQ GHVPLLSEKI IEFSDLLLEG INIVGCCCTG NDALMRHGIP
PADDDONK_03117  YGSQIPLEMG QDILFGTPQP HAVNVDLGII DPTYVNIAAN GHEPFIGVAL IKAAHKNQDG LHIIGSIETG QELIQRFPIG
AKAIOEBN_03183  WGGSMIATDI SDILFGTPAP ILGKANLGVL KQNMVNVIVH GHEPTLSQMI VAASQEVIEG INLSGICCTA NEILMRQGIP
ECJEKJPK_00295  ---------- ---------- ---------- ---------- --------MI VAATAEMQDG IQLSGICCTA NEILQRHGVP
ECJEKJPK_02254  ---------- ---------- ---------- ---------- ---------- ---------- ---------- ----------
EPCNEHLO_04340  WGGSMIATDI SDILFGTPAP ILGQANLGVL KQDMVNVVVH GHEPTLSEMI VAASQEIIEG VNLAGICCTA NEILMRQGIP
GLJNLELP_01063  YTGMSLATDL TDILFGTPEP VITEANLGVL DPEKVNIAVH GHNPLLSQMV VYAARELEGG INLVGICCTG NEVLMREGIP
HFBOOHMF_00942  WGGCMIATEL QDIMFGTPKP TAGRSNLGVI KKDHVNIIVH GHEPQLAEAI VLACNEVIKG IVLSGLCCTA NELLVRHGIP
```

```
                    ....|....| ....|....| ....|....| ....|....| ....|....| ....|....| ....|....| ....|....|
                          245        255        265        275        285        295        305        315
GIHMDANB_00018      QTGNWINQEY LIATGAIDLV MMDLNCSTPG LKNMADHFHT RVISVDKLVR MAGVTHLDPD EQAMQIVKMS IDAYKMRDSK
GIHMDANB_00023      LTGNWLSIEP ALATGGIDVL AMDMNCSPPG MAEYQDKYNT SLISVSKLIN VPGMKQIIPA AQAQKLIDIA IENFKKRKKT
PADDDONK_01837      QTGNWINQEY LIATGAIDLV MMDLNCSTPG LKNMADHFHT RVISVDKLVR MAGVTHLDPD EQAMQIVKMS IDAYKMRDSK
PADDDONK_01842      LTGNWLSIEP ALATGGIDVL AMDMNCSPPG MAEYQDKYNT SLISVSKLIN VPGMKQIIPA AQAQKLIDIA IENFKKRKKT
PADDDONK_02117      IIGSMSKELK YIRSGIPDVL VTDEQCVRAD ILKEAQKLHI PVIATNEKIT YGGVVLLDFE KIGELVPKLA MKMGPIRKEE
PADDDONK_03833      IAGSNIHQEL IIATGLAEAV VVDVQCIYPN IENVARHFHT KIISTMKEAR FKSAQHIPED DAAKSILNAA IENFPKRGHR
PADDDONK_03053      IAGSNIHQEL IIATGLAEAV VVDVQCIYPN IENVARHFHT KIISTMKEAR FKSAQHIPED DAAKSILNAA IENFPKRGHR
PADDDONK_03117      MIGNWLSVEP ALATGGIDVF AMDMNCSPPG MGEYQDKYNT NLISVSKLVN VPGMKQMIPA AQAQKLIDIA IESFKKRKKA
AKAIOEBN_03183      AAGNFLQQEL AILTGAVEAM VVDVQCIMQA LVGLAANFHT KIITTSPKVK IKGATHIEEL TIAKQILKAA IDNFQNR-GE
ECJEKJPK_00295      LCGTFLQQEL AIITGACDAM VVDIQCIFQN LANVAKCFHT KLITTHPIAR MENVIHIEEI DDAKQIVKLA IDNFQNRKTE
ECJEKJPK_02254      ---------- ---------- ---------- ---------- ---------- ---------- ---------- ----------
EPCNEHLO_04340      AAGNFLHQEL SILTGAVEAM VVDVQCIMQA LVKLAENFHT LVITTSPKVK IKGATHIEEL TIAKQILRTA IDNFKNR-GP
GLJNLELP_01063      TATNFGSQEL AIMTGVLDTM IMDVQCIAPG VMDVCNCFHT QLVSTSNISK VPGSYHVAEM EDARKIVNLA IKQYAKRKEL
HFBOOHMF_00942      MAGHMTIQEA AIATGAVEAM VVDIQCVMQS LAEAAKSFHT KLITTLSKAK IFGAEHIEEL ESAKKIVMVA IANYKNRDEK

                   ....|....| ....|....| ....|....| ....|....| ....|....| ....|....| ....|....| ....|....|
                          325        335        345        355        365        375        385        395
GIHMDANB_00018      VHIPKYKSEV MAGFSVESCK AALGGTWNPL IDAIKAGSVK GIVAVVGCTT AKTKHDVSVK LSRELIKRNI LVINAGCVSS
GIHMDANB_00023      TTPSSKKQNA VIGFSTEACL GALGGTLDPL LDVIKKGTLK GVVALVSCTT LRDGQDNTLK MARELIKRDI LVISAGCGNA
PADDDONK_01837      VHIPKYKSEV MAGFSVESCK AALGGTWNPL IDAIKAGSVK GIVAVVGCTT AKTKHDVSVK LSRELIKRNI LVINAGCVSS
PADDDONK_01842      TTPSSKKQNA VIGFSTEACL GALGGTLDPL LDVIKKGTLK GVVALVSCTT LRDGQDNTLK MARELIKRDI LVISAGCGNA
PADDDONK_02117      FDCPQSISDA MA----SLFE ILHDKEIREE GRNLVLGTTP GVLAFVGCGN YPDGKD-VYD IVEEMIQRSY IIITSGCSAM
PADDDONK_03833      IFLPQKSHDL MAGFSVENVL GVLAAPFKPL IESIKSGDIN GIVLMAGCIS PEMT-ESQVI IVKELLKQNV LVFSTGCSAQ
PADDDONK_03053      IFLPQKSHDL MAGFSVENVL GVLAAPFKPL IESIKSGDIN GIVLMAGCIS PEMT-ENQVI IVKELLKQNV LVFSTGCSAQ
PADDDONK_03117      TNPSSKKQNA VIGFSTEACL GALGGTLTPL LDVIKKGTLK GVVALVSCTT LRDSQDNTLK MARELIKRDI LVISAGCGNA
AKAIOEBN_03183      TKIPDVREDL IPGFSHEYIN YMLGGSFRPL NDAVMSGRIR GVAAIVGCNN PRSRQDLHTY VTQELLKQDV LVVETGCGAI
ECJEKJPK_00295      VMIPRQKAPM VAGFGVESIE YHLGGSYHPL NDNIINGRIR GIGGVVGCNN VRTRHNGHIQ VVKELIKNDV IVLTTGCNAI
ECJEKJPK_02254      ---------- MHGFSHEYIN YMLGGSYVPL NDNIINGRIR GVAGVVGCTN PRVKQDLHVE LVKELIKNDV LVLQTGCSQI
EPCNEHLO_04340      VQIPNARENL IPGFSHEYIN YMLGGSFRPL NDAIMTGRIR GVAAIVGCNN PRSTQDLHTY VVKELLKQDV LVVQTGCGAI
GLJNLELP_01063      VRIPEFKKQV VGGFSFESMM DLFGRPISVL TEAVMAGEIK GVCAFAGCNN QKTIHDSIIT IAKELAKNDV FMVSTGCAAG
HFBOOHMF_00942      VFIPSAEPNL VAGFSHETIK YMLGGSYRPL NDNIINGRIR GVVGIAGCTS PKAGVCSYIN LARELIANNF LVVATGCAAG

                    ....|....| ....|....| ....|....| ....|....| ....|....| ....|....| ....|....| ....|....|
                          405        415        425        435        445        455        465        475
GIHMDANB_00018      AIQIEGEDAG EGLKAVCRSL HIPPCLNYGS CVDIGRIGVA VTEIAAALGV DPSALPVAAS APEYLEQKAV ADGAFAVAFG
GIHMDANB_00023      ALQVGGEQAG PGLRAVCELL KIPPVLSFGT CTDTGRIALL VTAVADALGV DPSQLPVAVT APEYMEQKAT IDAIFALAFG
PADDDONK_01837      AIQIEGEDAG EGLKAVCRSL HIPPCLNYGS CVDIGRIGVA VTEIAAALGV DPSALPVAAS APEYLEQKAV ADGAFAVAFG
PADDDONK_01842      ALQVGGEQAG PGLRAVCELL KIPPVLSFGT CTDTGRIALL VTAVADALGV DPSQLPVAVT APEYMEQKAT IDAIFALAFG
PADDDONK_02117      DVGMFKDKDG KTLYERYPGR VKGNLLNTGS CVSNAHIAAT TIKVASEIDY VLNRVGAVGI AWGAYSQKAF AIGTGCNRLG
PADDDONK_03833      ACARGGDMAG DKLKGILRSL GLPPVWHFGS CVDNSRPIIL AIALAQKMGI SLKDMPVAAS ASDWVAEKAA AIGAGTVALG
PADDDONK_03053      ACARGGDMAG DKLKGILRSL GLPPVWHFGS CVDNSRPIIL AIALAQKMGI SLKDMPVAAS ASDWVAEKAA AIGAGTVALG
PADDDONK_03117      ALQVGGEQAG PGLRAVCELL KIPPVLSFGT CTDTGRITLL VTAVADALGV DPSQLPVAVT APEYMEQKAT IDALFALAFG
AKAIOEBN_03183      ASAKLGEDVG PGLREICETV GIPPVLHMGS CVDNTRILTV LTQMVAGLGD DIDEVPAVGL APEWMSEKAL SIGAYCVASG
ECJEKJPK_00295      ACAMEGEVCG PGLAEVCETV GIPPVLHMGS CVDNSRILLA AVEVVKGLGQ DISDLPAAGS APEWMSEKAI SIGHYFVA--
ECJEKJPK_02254      ALAKAGEHAG PGLKEVCETV GMPPVLGIGS CVDNSRILIA ASEMVKGLGN SIADLPVAGA APEYMSEKAI SIGQYFVASG
EPCNEHLO_04340      ASAKLGEDVG PGLREVCEAV GIPPVLHMGS CVDNTRILTV LTQMVEGLGD DIDQIPAVGL APEWMSEKAL SIGVYCVASG
GLJNLELP_01063      ALAKAGEDAG EGLKKFIKRL ELPLVFHMGS CVDNSRVANL WTAMAQNLNI SVPQLPFAAS APEAMSEKAI SIGSWVISMG
HFBOOHMF_00942      QCASDGEDCG QGLKEVCEAV GIPPVLHSGA CVDNSRILIA VSEMVKGLGN DISELPVAGA CTEWMSEKAI AIGQFLVASG

                    ....|....| ....|....| ....|....| ....|....| ....|....| ....|
                          485        495        505        515        525        535
GIHMDANB_00018      LLLHLAPVPP VTGAPLVAKV LTNDVESLTG GELDPVKAAD AIEAHINTKR KALGL
GIHMDANB_00023      LYTHVSPLPP VAGAPRLVKL LTEDLEGITG GETDMVDAAN GIEAHIMKKR AALGL
PADDDONK_01837      LLLHLAPVPP VTGAPLVAKV LTNDVESLTG GELDPVKAAD AIEAHINTKR KALGL
PADDDONK_01842      LYTHVSPLPP VAGAPRLVKL LTEDLEGITG GETDMVDAAN GIEAHIMKKR AALGL
PADDDONK_02117      IPVVTGPEPP ETKDELLAKL CFRELEKYLK -EADPVAKRE QLMKLLGEK- GWKDW
PADDDONK_03833      ITVHLGRAPP ILGGPEVVTL LTQKSEELFG AEEDPIKASK LLLLHIGKAR EKLGL
PADDDONK_03053      ITVHLGRAPP ILGGPEVVTL LTQKSEELFG AEEDPIKASK LLLLHIGKAR EKLGL
PADDDONK_03117      LYTHVAPMPP VTGAPRLVKL LTVDLEGITG GETDMVEAAK GIEAHIMKKR SALGL
AKAIOEBN_03183      AYVIFGGSSP VSGMPLVLHY LSEGWQEIYG GVADPNEMIQ RTLDHIDKKR AALGL
ECJEKJPK_00295      ---------- ---------- ---------- ---------- ---------- -----
ECJEKJPK_02254      VFTVFGVTFP TVEGTKFHKH LFEDLEKMGM GAIDPQEMAR LIIEHIDKKR KALGI
EPCNEHLO_04340      AYVIFGGSSP VSGMPAVLHY LTEGWEKLYG GDVDPDSMIQ KTLEHIDRKR TALGL
GLJNLELP_01063      IPCHVGVLPP IEGSELVYGV ATQIARDVFG GETDPVKASE KLLDRIEKRA WKLKI
HFBOOHMF_00942      IYTVFGMNSP VAGAPDMQRL LTKEMEEMCG AESDLKKIGK MLMDHIEKKR DALGI
```

Supplementary Figure 2. Average amino acid identity (AAI) matrix of the bifunctional carbon monoxide dehydrogenase CdhA and monofunctional CooS homologs from the '*Ca.* Methanoperedens BLZ2' genome and mobile genetic element. Percent identity matrix created by Clustal2.1 using default settings[1]. Source data are provided as a Source Data file.

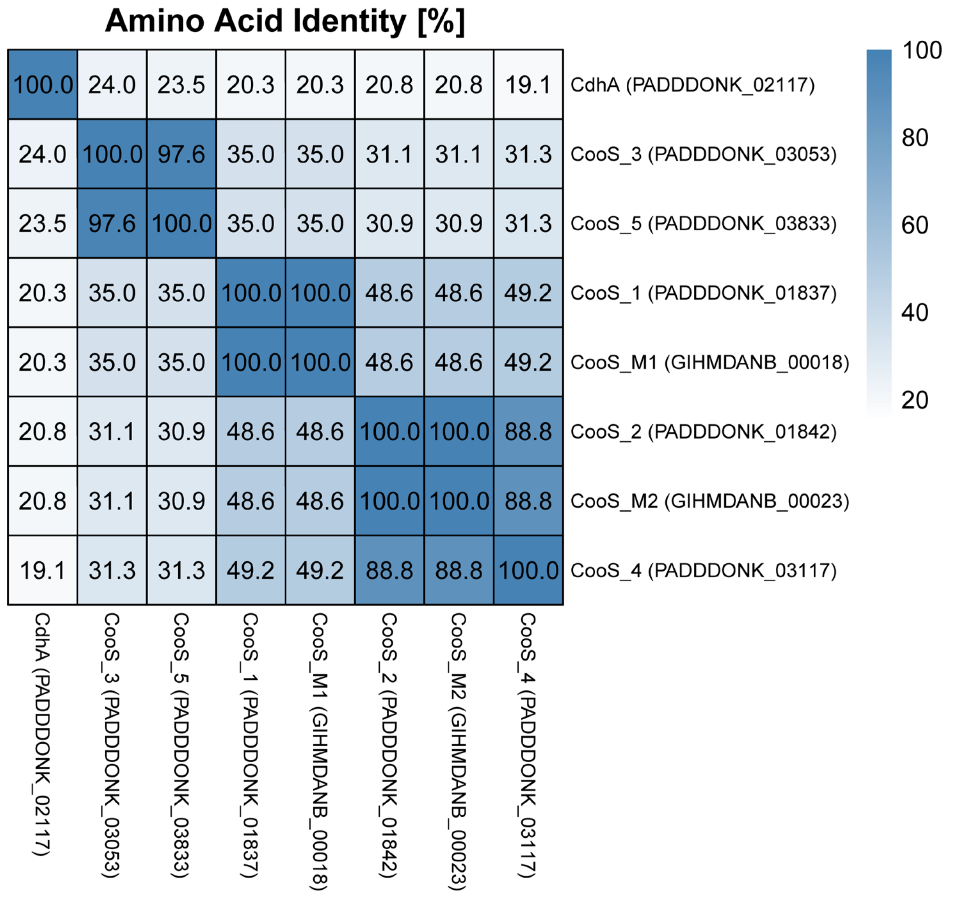

Supplementary Figure 3. Global proteome-based similarity of two closed *Methanoperedenaceae* genomes and associated mobile genetic elements The dendrogram was constructed using high-scoring segment pairs from DiGAlign v2.0-based protein alignments[2]. Included are previously identified MGEs associated to ANME and the closed genome of '*Ca*. Methanoperedens nitroreducens'[3–5]. Visualization was performed in iTOL v6.9.1[6], with mid-point rooting and branch lengths omitted for clarity.

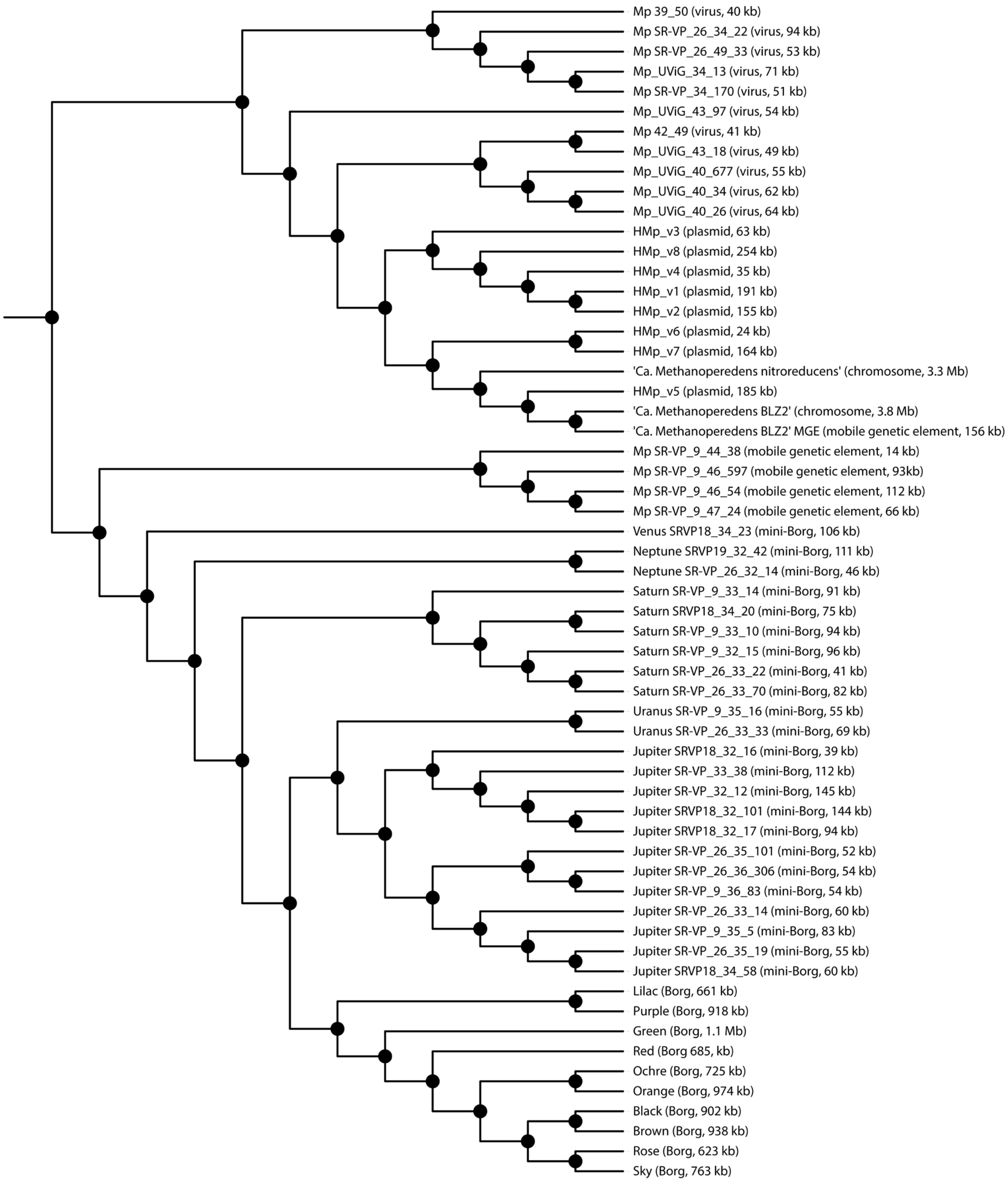

Supplementary Table 2. Identified tandem repeats on the mobile genetic element and prokaryotic viral orthologous groups (pVOGs). Detection of imperfect tandem repeats was performed using ETANDEM with default parameters from the EMBOSS suite (v5.0.0)[7]. The pVOGs were identified using Phigaro (v2.4.0) with default parameters[8].

Table 2.1. Identified imperfect tandem repeats on the mobile genetic element, the unique region spans from 64.5kb - 91.6kb.

Length	Start	End	Region	Unit size	Repeats	Match [%]	motif
33	88418	88489	Unique	9	8	79.2	ctatagatt
30	73778	73867	Unique	10	9	72.2	tttcataaat
29	83104	83223	Unique	15	8	68.3	aggtggtggatctg g
24	95401	95455	Shared	11	5	81.8	aagtattaatc
20	23781	23828	Shared	12	4	83.3	taaaaaagagga

Table 2.2. Identified pVOGs in the prophage region from 88756 until 98284 including identified integrase protein families (pfams).

Locus tag	pVOG	start	end	score	strand	Integrase pfams: (PF00589 and PF13495)
GIHMDANB_00092	VOG5768	88756	89211	0.000035	+	
GIHMDANB_00094	VOG5337	89866	90213	0.000096	+	
GIHMDANB_00095	VOG0275	90253	91149	3.2E-30	-	PF00589 and PF13495
GIHMDANB_00096	VOG7404	91257	91511	0.0024	-	
GIHMDANB_00097	VOG0275	91747	92940	3.8E-25	+	PF00589 and PF13495
GIHMDANB_00100	VOG0183	94761	94928	0.000066	+	
GIHMDANB_00101	VOG2124	95098	95763	0.000093	+	
GIHMDANB_00102	VOG2752	96056	96502	0.000017	+	
GIHMDANB_00103	VOG4632	96530	98284	1.9E-17	+	

Supplementary References

1. Larkin, M. A. *et al.* Clustal W and Clustal X version 2.0. *Bioinformatics* **23**, 2947–2948 (2007).
2. Nishimura, Y., Yamada, K., Okazaki, Y. & Ogata, H. DiGAlign: Versatile and Interactive Visualization of Sequence Alignment for Comparative Genomics. *Microbes Environ.* **39**, n/a (2024).
3. Al-Shayeb, B. *et al.* Borgs are giant genetic elements with potential to expand metabolic capacity. *Nature* **610**, 731–736 (2022).
4. McIlroy, S. J. *et al.* Anaerobic methanotroph 'Candidatus Methanoperedens nitroreducens' has a pleomorphic life cycle. *Nat. Microbiol.* **8**, 321–331 (2023).
5. Shi, L.-D. *et al.* Methanotrophic Methanoperedens archaea host diverse and interacting extrachromosomal elements. *Nat. Microbiol.* **9**, 2422–2433 (2024).
6. Letunic, I. & Bork, P. Interactive Tree of Life (iTOL) v6: recent updates to the phylogenetic tree display and annotation tool. *Nucleic Acids Res.* **52**, W78–W82 (2024).
7. Rice, P., Longden, I. & Bleasby, A. EMBOSS: The European Molecular Biology Open Software Suite. *Trends Genet.* **16**, 276–277 (2000).
8. Starikova, E. V. *et al.* Phigaro: high-throughput prophage sequence annotation. *Bioinformatics* **36**, 3882–3884 (2020).
