# Supplementary material for: Carbon monoxide metabolism in freshwater anaerobic methanotrophic archaea
Source: Nat Commun. 2026 Apr 14;17:3460. doi: 10.1038/s41467-026-70080-4 (PMC13079737; doi:10.1038/s41467-026-70080-4)
Supplement: Supplementary file 2 — Description of Additional Supplementary Files [file 41467_2026_70080_MOESM2_ESM.pdf]

Description of Additional Supplementary Files:

Supplementary Data 1: Overview of obtained Metagenome-Assembled Genomes (MAGs).

Supplementary Data 2: DNA based relative abundance of Metagenome-Assembled Genomes (MAGs).

Supplementary Data 3: Overview of metabolisms of interest of the dominant bacterial community based on KEGG orthologs (KO).

Supplementary Data 4: Measured substrate and product concentrations and calculated carbon balances under CO/CO2 conditions.

Supplementary Data 5: RNA based relative abundance of Metagenome-Assembled Genomes (MAGs).

Supplementary Data 6: Identified aerobic CODHs (Mo-CODHs) including average mapped reads under different conditions.

Supplementary Data 7: Identified anaerobic CODHs (Ni-CODHs) of different MAGs including CODH/ACS cluster overview. For each Ni-CODH the mapped transcripts and DESeq2 output are provided.

Supplementary Data 8: Overview of transcriptome raw count data and transcriptome results for the top five transcriptionally active community members, including volcano plots for '*Ca.* Methanoperedens BLZ2'.

Supplementary Data 9: Overview of central metabolism '*Ca.* Methanoperedens. BLZ2' including transcriptome results.

Supplementary Data 10: Phylogenetic classification of Ni-dependent carbon monoxide dehydrogenases (CODHs) of '*Ca*. Methanoperedens BLZ2' using GTDB R220 as well as against the earlier obtained mobile genetic elements. Black dots represent ≥ 90% support percentages from 1000 replicates. Clades depict the classification of CODHs into structural groups reflecting structural diversity and potential functional specialization.
