# Supplementary figures and images for: Carbon monoxide metabolism in freshwater anaerobic methanotrophic archaea
Source: Nat Commun. 2026 Apr 14;17:3460. doi: 10.1038/s41467-026-70080-4 (PMC13079737; doi:10.1038/s41467-026-70080-4)

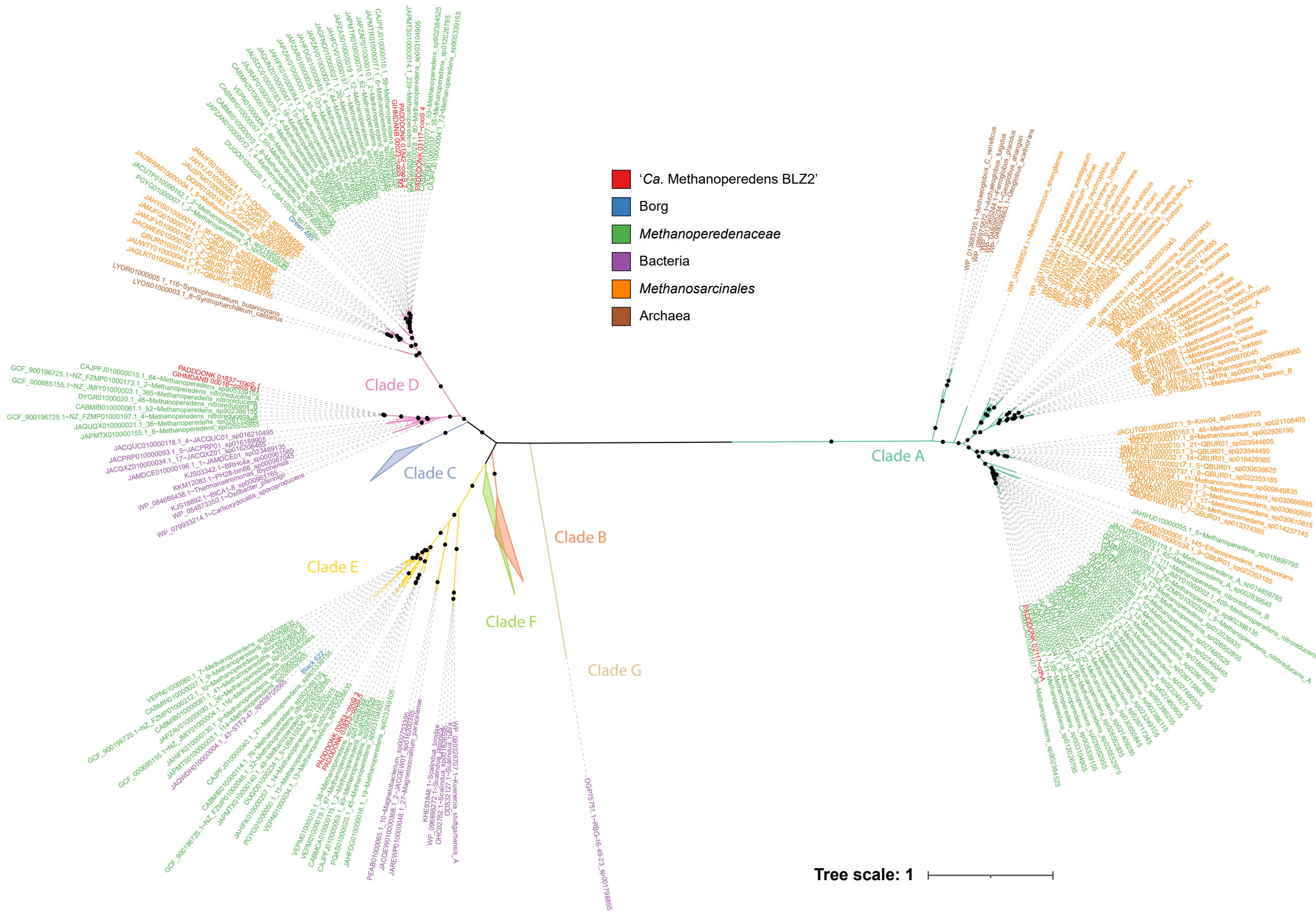

Supplement: Supplementary file 12 — Supplementary Dataset 10 [file 41467_2026_70080_MOESM12_ESM.pdf]
