# Supplementary material for: Carbon monoxide metabolism in freshwater anaerobic methanotrophic archaea
Source: Nat Commun. 2026 Apr 14;17:3460. doi: 10.1038/s41467-026-70080-4 (PMC13079737; doi:10.1038/s41467-026-70080-4)
Supplement: Supplementary file 13 — Reporting Summary [file 41467_2026_70080_MOESM13_ESM.pdf]

nature portfolio

Corresponding author(s): Cornelia Welte

Last updated by author(s): Feb 2, 2026

Reporting Summary

Nature Portfolio wishes to improve the reproducibility of the work that we publish. This form provides structure for consistency and transparency in reporting. For further information on Nature Portfolio policies, see our Editorial Policies and the Editorial Policy Checklist.

Statistics

For all statistical analyses, confirm that the following items are present in the figure legend, table legend, main text, or Methods section.

n/a	Confirmed	
[ ]	[x]	The exact sample size (*n*) for each experimental group/condition, given as a discrete number and unit of measurement
[ ]	[x]	A statement on whether measurements were taken from distinct samples or whether the same sample was measured repeatedly
[ ]	[x]	The statistical test(s) used AND whether they are one- or two-sided<br>*Only common tests should be described solely by name; describe more complex techniques in the Methods section.*
[x]	[ ]	A description of all covariates tested
[x]	[ ]	A description of any assumptions or corrections, such as tests of normality and adjustment for multiple comparisons
[ ]	[x]	A full description of the statistical parameters including central tendency (e.g. means) or other basic estimates (e.g. regression coefficient) AND variation (e.g. standard deviation) or associated estimates of uncertainty (e.g. confidence intervals)
[ ]	[x]	For null hypothesis testing, the test statistic (e.g. *F*, *t*, *r*) with confidence intervals, effect sizes, degrees of freedom and *P* value noted<br>*Give P values as exact values whenever suitable.*
[x]	[ ]	For Bayesian analysis, information on the choice of priors and Markov chain Monte Carlo settings
[x]	[ ]	For hierarchical and complex designs, identification of the appropriate level for tests and full reporting of outcomes
[x]	[ ]	Estimates of effect sizes (e.g. Cohen's *d*, Pearson's *r*), indicating how they were calculated

Our web collection on statistics for biologists contains articles on many of the points above.

Software and code

Policy information about availability of computer code

Data collection	For data collection, no software was used.
Data analysis	Only publicly available software and code was used for Data analysis in this study; please find the respective papers or GitHub referrals below. Figures were made using a combination of the software mentioned below and Adobe Illustrator 2025.<br><br>Publicly available software generic use.<br>R version 4.4.0 (DESeq2, figure generation)<br>Jupyterlab 4.2.5 (motif searches such as aerobic CODHs and multiheme cytochromes)<br><br>Filtlong v0.2.1; (https://github.com/rrwick/Filtlong).<br>Flye v2.8.3; Kolmogorov, M. et al. metaFlye: scalable long-read metagenome assembly using repeat graphs. Nat. Methods 17, 1103–1110 (2020).<br>Bandage v0.8.1; Wick, R. R., Schultz, M. B., Zobel, J. & Holt, K. E. Bandage: Interactive visualization of de novo genome assemblies. Bioinformatics 31, 3350–3352 (2015).<br>Racon v1.4.21; Vaser, R., Sović, I., Nagarajan, N. & Šikić, M. Fast and accurate de novo genome assembly from long uncorrected reads. Genome Res. 27, 737–746 (2017).<br>Pilon v1.2.4; Walker, B. J. et al. Pilon: An Integrated Tool for Comprehensive Microbial Variant Detection and Genome Assembly Improvement. PLOS ONE 9, e112963 (2014).<br>dRep v3.4.2; Olm, M. R., Brown, C. T., Brooks, B. & Banfield, J. F. dRep: a tool for fast and accurate genomic comparisons that enables improved genome recovery from metagenomes through de-replication. ISME J. 11, 2864–2868 (2017).

CheckM2 v1.1.0; Chklovski, A., Parks, D. H., Woodcroft, B. J. & Tyson, G. W. CheckM2: a rapid, scalable and accurate tool for assessing microbial genome quality using machine learning. Nat. Methods 20, 1203–1212 (2023).
CoverM v0.7.0; Aroney, S. T. N. et al. CoverM: read alignment statistics for metagenomics. Bioinformatics 41, btaf147 (2025).
Prokka v1.14.6; Seemann, T. Prokka: Rapid prokaryotic genome annotation. Bioinformatics 30, 2068–2069 (2014).
EggNOG-mapper v2.1.12; Cantalapiedra, C. P., Hernández-Plaza, A., Letunic, I., Bork, P. & Huerta-Cepas, J. eggNOG-mapper v2: Functional Annotation, Orthology Assignments, and Domain Prediction at the Metagenomic Scale. Mol. Biol. Evol. 38, 5825–5829 (2021).
Huerta-Cepas, J. et al.
EggNOG v5.0; EggNOG 5.0: A hierarchical, functionally and phylogenetically annotated orthology resource based on 5090 organisms and 2502 viruses. Nucleic Acids Res. 47, D309–D314 (2019).
KofamScan v1.3.0; Aramaki, T. et al. KofamKOALA: KEGG Ortholog assignment based on profile HMM and adaptive score threshold. Bioinformatics 36, 2251–2252 (2020).
Proksee; roksee Grant, J. R. et al. Proksee: in-depth characterization and visualization of bacterial genomes. Nucleic Acids Res. 51, W484–W492 (2023).
DigAlign v2.0; Nishimura, Y., Yamada, K., Okazaki, Y. & Ogata, H. DiGAlign: Versatile and Interactive Visualization of Sequence Alignment for Comparative Genomics. Microbes Environ. 39, n/a (2024).
iTol v6.9.1; Letunic, I. & Bork, P. Interactive Tree of Life (iTOL) v6: recent updates to the phylogenetic tree display and annotation tool. Nucleic Acids Res. 52, W78–W82 (2024).
Phigaro v2.4.0; Starikova, E. V. et al. Phigaro: high-throughput prophage sequence annotation. Bioinformatics 36, 3882–3884 (2020).
EMBOSS v5.0.0; Rice, P., Longden, I. & Bleasby, A. EMBOSS: The European Molecular Biology Open Software Suite. Trends Genet. 16, 276–277 (2000).
Mafft v7.525; Katoh, K. & Standley, D. M. MAFFT Multiple Sequence Alignment Software Version 7: Improvements in Performance and Usability. Mol. Biol. Evol. 30, 772–780 (2013).
TrimAl v1.5.0; Capella-Gutiérrez, S., Silla-Martínez, J. M. & Gabaldón, T. trimAl: a tool for automated alignment trimming in large-scale phylogenetic analyses. Bioinformatics 25, 1972–1973 (2009).
GTDB-Tk v2.4.0; Chaumeil, P. A., Mussig, A. J., Hugenholtz, P. & Parks, D. H. GTDB-Tk: A toolkit to classify genomes with the genome taxonomy database. Bioinformatics 36, 1925–1927 (2020).
Diamond v2.1.13; Buchfink, B., Reuter, K. & Drost, H.-G. Sensitive protein alignments at tree-of-life scale using DIAMOND. Nat. Methods 18, 366–368 (2021).
Muscle v5.0.1278; Edgar, R. C. Muscle5: High-accuracy alignment ensembles enable unbiased assessments of sequence homology and phylogeny. Nat. Commun. 13, 6968 (2022).
VeryFastTree v4.0.5; Piñeiro, C., Abuín, J. M. & Pichel, J. C. Very Fast Tree: speeding up the estimation of phylogenies for large alignments through parallelization and vectorization strategies. Bioinformatics 36, 4658–4659 (2020).
Aviary v0.9.0 (https://github.com/rhysnewell/aviary).

For manuscripts utilizing custom algorithms or software that are central to the research but not yet described in published literature, software must be made available to editors and reviewers. We strongly encourage code deposition in a community repository (e.g. GitHub). See the Nature Portfolio guidelines for submitting code & software for further information.

Data

Policy information about availability of data

All manuscripts must include a data availability statement. This statement should provide the following information, where applicable:

- Accession codes, unique identifiers, or web links for publicly available datasets
- A description of any restrictions on data availability
- For clinical datasets or third party data, please ensure that the statement adheres to our policy

The metagenomic and metatranscriptomic data have been deposited in the European Nucleotide Archive database under study accession https://www.ebi.ac.uk/ena/browser/view/PRJEB97457, with identifiers: SAMEA120155624, SAMEA120631852 and SAMEA120155615-120155623. The metagenome-assembled genomes (MAGs) generated in this study are under the same study accession, with identifiers SAMEA121075817-SAMEA121075852. The assembled mobile genetic element is part of SAMEA121075817. The full non-redundant set of MAGs including associated metadata, has also been deposited on Figshare (https://doi.org/10.6084/m9.figshare.30903899). Source Data are provided with this paper.

Research involving human participants, their data, or biological material

Policy information about studies with human participants or human data. See also policy information about sex, gender (identity/presentation), and sexual orientation and race, ethnicity and racism.

Reporting on sex and gender	Not applicable
Reporting on race, ethnicity, or other socially relevant groupings	Not applicable
Population characteristics	Not applicable
Recruitment	Not applicable
Ethics oversight	Not applicable

Note that full information on the approval of the study protocol must also be provided in the manuscript.

Field-specific reporting

Please select the one below that is the best fit for your research. If you are not sure, read the appropriate sections before making your selection.

- [x] Life sciences
- [ ] Behavioural & social sciences
- [ ] Ecological, evolutionary & environmental sciences

For a reference copy of the document with all sections, see nature.com/documents/nr-reporting-summary-flat.pdf

Life sciences study design

All studies must disclose on these points even when the disclosure is negative.

Sample size

No formal sample size calculation was performed. Sample sizes were determined based on experimental feasibility, biomass availability, and established practice in anaerobic microbial physiology studies. For physiological batch activity assays, biological duplicates, triplicates, or quadruplicates were used depending on available biomass for each experimental condition. These replicate numbers were sufficient to assess reproducibility of quantitative gas and metabolite measurements.

Transcriptomic analyses were performed using biological triplicates, which is standard for differential gene expression analysis. Metagenomic sequencing was performed on a single bioreactor sample, as the same continuously operated enrichment reactor was used as the inoculum source for all batch activity assays. Metatranscriptomic sequencing was performed at a defined time point (16 hours into the assay) during a batch activity assay to capture transcriptional responses under the tested condition.

Data exclusions

No data were excluded

Replication

Physiological data are reproducable in terms of relative trends, absolute rates can differ slightly because of the amount of biomass in the bioreactor and community composition. For comparative experiments all experiments were done as fast as time allowed ensuring minimal difference across tested conditions. All rates are reported and displayed in the paper.

Randomization

no randomization was done in this study, biomass derived from a single continuously stirred sequential fed-batch bioreactor so equal distribution of biomass across all tested conditions

Blinding

Blinding was not performed in this study. Investigators were responsible for reactor and culture maintenance as well as experimental execution and data analysis. Blinding was not relevant, as group allocation was defined by experimental conditions (substrate and electron acceptor availability) and outcome measures were quantitative and instrument-based.

Reporting for specific materials, systems and methods

We require information from authors about some types of materials, experimental systems and methods used in many studies. Here, indicate whether each material, system or method listed is relevant to your study. If you are not sure if a list item applies to your research, read the appropriate section before selecting a response.

Materials & experimental systems

n/a	Involved in the study
x	Antibodies
x	Eukaryotic cell lines
x	Palaeontology and archaeology
x	Animals and other organisms
x	Clinical data
x	Dual use research of concern
x	Plants

Methods

n/a	Involved in the study
x	ChIP-seq
x	Flow cytometry
x	MRI-based neuroimaging

Plants

Seed stocks

not applicable, no seed stocks were used.

Novel plant genotypes

not applicable, no plants were used.

Authentication

not applicable, no plants or seed stocks were used.
